# Genome-wide analysis of the *Pleurotus eryngii* laccase gene (*PeLac*) family and functional identification of *PeLac5*

**DOI:** 10.1186/s13568-023-01608-w

**Published:** 2023-09-28

**Authors:** Zihao Li, Yuanyuan Zhou, Congtao Xu, Jinlong Pan, Haikang Li, Yi Zhou, Yajie Zou

**Affiliations:** 1 State Key Laboratory of Efficient Utilization of Arid and Semi-arid Arable Land in Northern China, Beijing, 100081 China; 2 Institute of Agricultural Resources and Regional Planning, Chinese Academy of Agricultural Sciences, Beijing, 100081 China

**Keywords:** *Pleurotus eryngii*, Laccase, Genome-wide profiling, Expression pattern, Lignocellulose degradation

## Abstract

The laccase gene family encodes multiple isozymes that are crucial for the degradation of substrates and the regulation of developmental processes in fungi. *Pleurotus eryngii* is an important edible and medicinal fungus belonging to the Basidiomycota phylum and can grow on a variety of natural substrates. In the present study, genome-wide profiling of *P. eryngii* identified 10 genes encoding its laccase isoenzymes. Conservative sequence analysis demonstrated that all *PeLac*s possess classical laccase structural domains. Phylogenetic analysis yielded four major subgroups, the members of which are similar with respect to conserved gene organization, protein domain architecture, and consensus motifs. The 10 *Pe*Lacs formed three groups together with 12 *Po*Lacs in *Pleurotus ostreatus*, indicating that they share a high level of evolutionary homology. Cis-responsive element analysis implied that *PeLac*s genes play a role in growth and development and lignocellulose degradation. Targeted overexpression of *PeLac5* reduced the time to primordia formation and their development to fruiting bodies. Gene expression patterns in the presence of different lignocellulosic substrates indicate that three *PeLac*s genes (*2*, *4*, and *9*) are key to lignocellulose degradation. This work presents the first inventory of laccase genes in *P. eryngii* and preliminarily explores their functions, which may help to uncover the manner by which these proteins utilize substrates.

**Supplementary Information:**

The online version contains supplementary material available at 10.1186/s13568-023-01608-w.

## Key Points

- *Pleurotus eryngii* genome uncovered 10 genes encoding laccase isoenzymes.
- Targeted overexpression of *PeLac5* reduced the time to primordia formation and their development to fruiting bodies.
- Three *PeLac*s genes (*2*, *4*, and *9*) are key to lignocellulose degradation.

**Supplementary Information:**

The online version contains supplementary material available at 10.1186/s13568-023-01608-w.

## Introduction

Over a long period of time, a wide range of microorganisms have evolved to synthesize cellulolytic enzymes [β-glucosidase (BGL), cellobiohydrolase (exo-glucanase or CBH), and endo-glucanase (EG)] and ligninolytic enzymes [e.g., lignin peroxidase (LiP), laccase, manganese peroxidase (MnP), and versatile peroxidase (VP)] that function synergistically to degrade lignocellulosic biomass (Lombard et al. 2013; Manavalan et al. 2015). However, only a few fungal groups, such as white-rot, brown-rot, and soft-rot fungi, possess the ability to effectively degrade lignocellulosic biomass. In particular, white-rot fungi including *Lentinula edodes*, *Pleurotus sajor-caju*, *Pleurotus ostreatus*, *Pleurotus eryngii*, *Flammulina velutipes*, and *Trametes versicolor* are capable of degrading both cellulose and lignin biopolymers (Manavalan et al. 2015) due to their capacity to secrete a complete system of extracellular lignocellulose-degrading enzymes that interact synergistically to facilitate the use of lignocellulose by fungi.

Laccase (p-diphenol:dioxygen oxidoreductase, EC 1.10.3.2), a blue multicopper oxidase (MCO), is capable of catalyzing the oxidation of many aromatic compounds while reducing molecular oxygen to water. It was first discovered from the tree *Rhus vernicifera* by Yoshida (1883), and was subsequently universally isolated from plants, fungi (*Ascomycetes*, *Basidiomycetes*), arthropods, and bacteria (Giuliano et al. 1988; Alexandre and Zhulin 2000; Brijwani et al. 2010; Giardina et al. 2010). Fungi laccases play a variety of critical physiological roles in lignocellulose degradation, morphogenesis, pigment synthesis, sporulation processes soil organic matter cycling and fungal–host interactions (Thurston 1994; Gianfreda et al. 1999; Sharma et al. 2007). Laccases and relevant MCOs are synthesized by most ligninolytic wood-rotting Basidiomycetes (Baldrian 2006) and have attracted substantial attention with respect to the enzymatic degradation of wood, since these fungi are the most productive decomposers of lignin (Wang et al. 2015). Laccase is uniquely superior in the degradation of lignocellulose because it does not require the involvement of H_2_O_2_ (Manavalan et al. 2015). Gene families encoding laccases are expressed extensively in various Basidiomycetes such as, *Ganoderma lucidum*, *Volvariella volvacea*, *P. sajor-caju* and *F. velutipes* (Soden and Dobson 2001; Liu et al. 2012; Bao et al. 2013; Wang et al. 2015), with the largest number (up to 17) found in *Coprinopsis cinerea* (Kilaru et al. 2006).

*Pleurotus eryngii*, a white-rot fungus that belongs to basidiomycetes and is also known as the king oyster mushroom, can be cultivated on a plethora of lignocellulose-based agricultural wastes (Parenti et al. 2013). *P. eryngii* is widely grown throughout the world due to its remarkable flavor, high nutritional value, and health benefits; however, its growth cycle is lengthy and limits its production. Shortening the growth cycle would significantly reduce costs; therefore, gene function investigation and bioinformatics of the laccase family in *P. eryngii* are critical to unraveling the transcriptional regulatory mechanisms and functions of the *PeLac* family, which may be valuable for breeding fast-growing strains and improving substrate utilization. Here, whole-genome sequencing of the *P. eryngii* ACCC52611 strain uncovered 10 laccase genes, which we named *PeLac1–10*. In the preliminary cultivation experiments, *PeLac5* was stably expressed at all developmental stages; especially during the mycelium and fruiting body stages, where its expression was increased by 25*–*40-fold, which was significantly higher than that of other laccase isozyme genes during the same period, indicating that it may play key roles in lignocellulose degradation, morphological construction, and other physiological activities (Li et al. 2021).

In the present study, genome-wide analysis of *PeLac*s was performed in *P. eryngii* 52,611 (Li et al. 2021) and their gene structure, systematic evolutionary pattern, amino acid sequences, and physicochemical properties were assessed. Overexpression of *PeLac5* increased enzyme activity 1.4–2.4-fold over WT, while targeted silencing of *PeLac5* reduced the time to primordia formation and their development to fruiting bodies, which indicates that *PeLac5* was demonstrated to positively regulate vegetative growth, being required for the formation of primordia and their development into fruiting bodies, in addition to exerting degradation activity in *P. eryngii*. Furthermore, the relative expression patterns of the 10 laccase genes were determined following growth on different substrates. These data further our understanding of the function of laccases in white-rot fungi and aid the exploration of the correlation between *Lac* family genes and lignocellulose degradation by *P. eryngii*.

## Materials and methods

### Culture of fungal strains and extraction of genomic DNA

*P. eryngii* (ACCC52611) was obtained from the Agricultural Culture Collection of China (ACCC), Institute of Agricultural Resources and Regional Planning, Chinese Academy of Agricultural Science. *P. eryngii* mycelia were cultivated on PDA in the dark at 25 ^o^C for 10 days, collected using a spoon, and frozen in liquid nitrogen. Genomic DNA was obtained by phenol, chloroform, and isoamyl alcohol extraction.

### Genome sequencing, assembly, and annotation

*P. eryngii* strain 52,611 DNA was de novo sequenced at Beijing Meining Biotechnology Co. Ltd. (Beijing, China). Genome assembly was carried out using Canu v1.8 and Smartdenovo (https://github.com/ruanjue/smartdenovo) to obtain two rough assemblies, and Quickmerge v0.3 (https://github.com/mahulchak/quickmerge) was used to fuse the two rough assemblies together to form the merged assembly. Subsequently, the merged assembly was compared with the filtered Illumina sequencing data and corrected using Pilon v1.23 (Walker et al. 2014) to obtain the final assembly. Gene model prediction was performed using the MAKER v2.3.1 (Cantarel et al. 2008) gene prediction software. The CDS (coding sequence) and protein obtained from the annotation of the JGI publicly available *P. eryngii* genome (https://mycocosm.jgi.doe.gov/Pleery1/Pleery1.home.html) were used as EST and protein evidence. Ab initio prediction was carried out using the Augustus software (Stanke et al. 2006) with *Laccaria bicolor* as the reference model. The initial prediction was optimized by retraining SNAP to obtain the final prediction results. The predicted gene sets were compared with the NCBI nr database, and functional annotation was performed using InterProScan v5.27-66.0 and Blast2 GO v5.2.5 (Conesa et al. 2005; Jones et al. 2014).

### Lac family gene identification and structural analysis

Members of the laccase family are known to possess Cu-oxidase, Cu-oxidase_2, and Cu-oxidase_3 (PF07732, PF00394, and PF07731) domains; therefore, the *P. eryngii* genome was searched using the HAMMER software (Finn et al. 2011). Genes encoding proteins that contain these three domains were classified as *Lac* family members, and were subsequently named using the prefix Pe for *P. eryngii* (i.e., *PeLac*) and numbered with respect to their genomic position. *P. eryngii* 52,611 genome was deposited into public database and are available at the following URL: http://www.gpgenome.com/species/41180. The accession numbers for the 10 laccase genes are listed in Additional file 1: Table S1.

### Analysis of protein sequences and domains

InterProScan 91.0 (https://www.ebi.ac.uk/interpro/search/sequence/) was used to identify the copper centers of laccase proteins. The SignalP 5.0 server (https://services.healthtech.dtu.dk/service.php?SignalP-5.0) predicted signal peptides and TMHMM-2.0 (https://services.healthtech.dtu.dk/service.php?TMHMM-2.0) predicted transmembrane topology. NetNGlyc-1.0 (https://services.healthtech.dtu.dk/service.php?NetNGlyc-1.0) was used to identify variable *N*-glycosylation sites. SOPMA (http://npsa-pbil.ibcp.fr/cgi-bin/npsa_automat.pl?page=/ NPSA/npsa_sopma.html) was used to evaluate the secondary structures of the laccase proteins.

### Alignment of protein sequences and phylogenetic analysis

After sequence alignment using Cluster X 1.83 (Larkin et al. 2007), GeneDoc 2.7 (Ge et al. 2022) was employed to visualize the protein sequences. Phylogenetic tree construction using the neighbour-joining method of MEGA 5.0 (Wang et al. 2015).

### Analysis of promoter cis-regulatory elements and motifs

Promoter cis-regulatory elements were predicted using the Plant Care server (http://bioinformatics.psb.ugent.be/webtools/plantcare/html/). The MEME website (https://meme-suite.org/meme/tools/meme) was used to predict conserved motifs of *Lac* proteins; the maximum quantity of motifs was set to 10. TBtools V1.105 (Chen et al. 2020) was employed to visualize cis-regulatory elements and motifs.

### Modeling of the tertiary structure of Lac proteins

SWISS-MODEL (https://swissmodel.expasy.org/) was employed to predict the tertiary structure of *Pe*Lac proteins by homology modeling, which was subsequently visualized using VMD v1.9.3 (Fernandes et al. 2019). The copper ions, secondary structure, and protein surface were displayed. Images were graphed using GLSL in Rendermodel; the visualization files of the 10 proteins were named *Pe*Lac1–10.vmd.

### Vector construction and agrobacterium*-*mediated transformation

The original C01-Mnsod1 vector (Hou et al. 2019) was engineered to induce the expression of *hyp* (the hygromycin phosphotransferase gene) under the control of a *Lac* promoter (Additional file 1: Fig. S1). Subsequently, *PeLac5* was obtained by PCR amplification of *P. eryngii* genomic DNA and inserted into this vector using *Spe*I and *pme*I (Takara, Tokyo, Japan). The resulting plasmid, OE-*PeLac5*, was subjected to DNA sequencing. Sense and antisense RNAi amplicons were generated by PCR and used to create the silencing plasmid. Finally, *P. eryngii* was transformed with *A. tumefacien*s GV3101 carrying the overexpression and silencing plasmids using a previously described method (Hou et al. 2019). The primers used for PCR are listed in Additional file 1: Table S2.

### Enzymatic activity assay

Transformant strains expressing WT *PeLac5* (OE-*PeLac5*.1 and OE-*PeLac5*.2) and RNAi targeted toward *PeLac5* (RNAi-*PeLac5*.17 and RNAi-*PeLac5*.20) were cultured on PDA plates containing 0.4 g/L naphthofen for 7 days at 25 °C. Coloration test of naphthofen-PDA plates was measured on days 3, 5, and 7. To determine the enzymatic activity of the fermentation solutions, CYM (solid complete yeast medium) liquid medium (glucose 20 g/L, peptone 2 g/L, KH_2_PO_4_ 1 g/L, MgSO_4_ 0.5 g/L) was inoculated with the strains and incubated at 25 °C with shaking for 15 days in the dark. Samples were extracted at 2*–*3-day intervals and centrifuged for 15 min at 12,000 × *g*. The enzymatic activity of the collected supernatants was determined using a previously described method (Nikolaivits et al. 2021).

### Production of mushrooms and measurement of the growth rate

The fungal strains were cultured on sawdust medium in the dark at 25 °C. The medium had a moisture content of 68% and a dry ingredient content of 32%, which consisted of 27.6% sawdust, 22.3% wheat bran, 18.9% corn cobs, 11% soya meal powder, 11% corn flour, 7.2% bagasse, 1.12% CaCO_3_, and 0.88% CaO. The mycelia were cultivated until they expanded beyond the bottle (30 days), then they were transferred to a room maintained under the following conditions to stimulate primordia formation: 15 ± 1 °C; 90–95% humidity; 12*–*14 h of light per day at an intensity of 300 lx during the button period; and a CO_2_ concentration of 1000–1500 ppm during the mycelium to primordium stage and 1500–3000 ppm during the young mushroom to fruiting body stage. To determine the growth rate, the same substrate was used with 80 g cultivated material in each of three tubes. The growth rate was recorded every 3 days and the average of the three tubes was taken.

### RNA extraction, cDNA synthesis, RT-qPCR, and analysis of gene expression

Mycelia were cultivated on solid CYM (solid complete yeast medium) and replacement media (equal quantities of agricultural waste such as wood chips of *Populus simonii*, *Castanea mollissima*, and *Pyrus bretschneideri* were used to replace the glucose in CYM media; the other ingredients remained the same) at 25 °C for 14 days. Subsequently, mycelia were collected with a spoon and frozen in liquid nitrogen. An Omega E.Z.N.A.® Fungal RNA Kit (Omega Bio-Tek, Norcross, GA, USA) was used to extract total RNA, and cDNA synthesis was carried out using a HiScript® III 1st Strand cDNA Synthesis Kit (+ gDNA wiper) (Vazyme, Nanjing, China). Sequences of the primers for the 10 *PeLac* genes and the reference gene are provided in Additional file 1: Table S1. A Quantitative Real Time PCR System (Applied Biosystems, USA) and Taq Pro Universal SYBR qPCR Master Mix (Vazyme, Nanjing, China) were used for RT-qPCR. Single product amplification was verified via the generation of a dissociation curve. Transcript levels were normalized to those of *PeGpd* (3- Phosphoglyceraldehyde dehydrogenase, *P. eryngii* genome Gene ID: HQ844045.1) and quantitated using the 2^−ΔΔCT^ method. Experimental data pertaining to three technical replicates performed in triplicate were analyzed.

## Results

### *P. eryngii* laccase gene family

A multigene family consisting of 10 laccases was discovered in the *P. eryngii* genome, the members of which were named *PeLac1–10*. The encoded *PeLac* proteins were classified as multicopper oxidases (Cu-oxidase_1, IPR001117; Cu-oxidase_2, IPR011706; and Cu-oxidase_3, IPR011707) by InterProScan. Essential information regarding these 10 *Pe*Lacs is provided in Table 1. All 10 laccases possess a signal peptide but no transmembrane regions according to analysis using SignalP (Petersen et al. 2011) and TMHMM2.0 (Xu et al. 2007), suggesting that these are secretory proteins. In addition, variable *N*-glycosylation sites were predicted in all laccases, the position of these sites within the folded enzymes will play a role of whether they can be used at all in glycosylation by being open to the environment. Secondary structure analysis revealed that laccases constitute 8.44*–*22.66% α-helices, 25.14*–*30.91% extended strands, 6.27*–*8.91% β-turns, and 43.29*–*54.41% random coils. There were no significant differences among the secondary structures of these proteins, with the exception of *Pe*Lac2 (Table 2).

**Table 1:** Characteristics of the *P. eryngii* laccase

Laccase	Signal peptide	Transmembrance topology	Number of potential N-glycosylation site
*Pe*Lac1	Y	N	1
*Pe*Lac2	Y	Y	1
*Pe*Lac3	Y	N	3
*Pe*Lac4	Y	N	4
*Pe*Lac5	Y	N	5
*Pe*Lac6	Y	N	1
*Pe*Lac7	Y	N	1
*Pe*Lac8	Y	N	3
*Pe*Lac9	Y	N	3
*Pe*Lac10	Y	N	4

**Table 2:** The predicted secondary structures of *Pe*Lac1–*Pe*Lac10 in *P. eryngii*

laccase	α-Helix (%)	Extended strand (%)	β-turn (%)	Random coils (%)
*Pe*Lac1	11.84	29.32	7.14	51.69
*Pe*Lac2	22.66	25.14	8.91	43.29
*Pe*Lac3	10.51	29.64	6.94	52.91
*Pe*Lac4	10.91	30.91	7.09	51.09
*Pe*Lac5	10.57	29.57	6.27	53.58
*Pe*Lac6	8.44	29.64	7.50	54.41
*Pe*Lac7	10.72	30.31	7.58	51.39
*Pe*Lac8	8.71	30.69	7.72	52.87
*Pe*Lac9	9.98	29.56	6.72	53.74
*Pe*Lac10	11.00	29.61	7.45	51.95

### Phylogeny and motifs of *PeLacs*

**Fig. 1:**
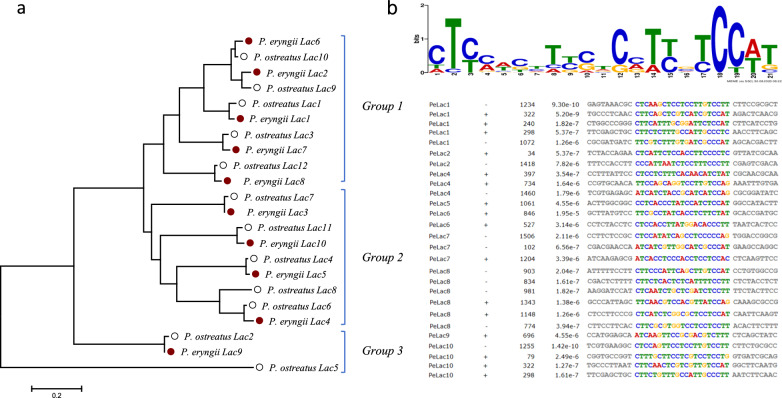
Phylogeny and motifs of *P. eryngii* laccases. **a** Neighbour joining tree of the Laccase amino sequences of *P. eryngii* and *P. ostreatus*. The tree was caculated using MEGA v7.0 (Kumar 2003) based on a Clustal X alignment. Bootstrap values are from 1000 replications. The scale bar indicates representing 0.2 amino acid substitutions per site. The red filled circles represent *P. eryngii* laccases and the black circles represent *P. ostreatus* laccases. **b** Schematic of the motif composition of the 10 *PeLac* genes. The logos above represent how conserved the motif is at each position. The lower sequence then shows potential transcription factor binding sites

In the phylogenetic tree, the amino acid sequences of laccase from *P. eryngii* and *P. ostreatus* were divided into three clades (Fig. 1a), for every *P. eryngii* laccase, there is a *P. ostreatus* laccase that is highly evolutionarily similar to it. *PeLac9* is similar to *PoLac2* and differentiates at an earlier stage, while *PeLac2 PeLac4, PeLac5* and *PeLac6* diverged later, suggesting that they may have evolved to perform more sophisticated functions. This similarity and difference in evolutionary relationships and gene structure predicts a possible diversity of laccase functions. The concept of motif was defined as the phenomenon where a certain pattern of connectivity occurs significantly more often in a complex network than in a random network (Henry et al. 2009). The use of motif analysis can be mined for transcription factor binding or DNA and RNA methylation modifications, which has implications for subsequent gene research. The MEME program was used to analyze the 10 *PeLac* sequences, which revealed 5 conserved motifs. Based on the E-value (E < 0.05) screening, we finally focused on a motif related to transcription factor activity (Fig. 1b). *PeLac8* contains six possible transcription factor binding sites, whereas no possible binding sites are predicted in *PeLac3*, *PeLac5* and *PeLac9* both contain only one possible binding site, and all other laccases have at least 2–5 possible binding sites. This suggests that there may be some commonality or diversity in the regulation of these genes by transcription factors,.indicating that these *PeLac* genes may perform different functions.

### Multiple sequence alignments and phylogenetic analysis

The sequences of the 10 laccase proteins were aligned to uncover conserved structural domains (Additional file 1: Fig. S2). All 10 identified laccases in *P. eryngii* contain three structural domains (I–III), which are also conserved within laccases of other fungi. Domain II is considered the MCO (second cupredoxin domain), and domains I and III are its N- and C-terminus, respectively. Together, these three domains form a structure that is consistent with the typical characteristics of laccase enzymes, indicating a common mechanism of copper oxidation and oxygen reduction.

A phylogenetic tree was constructed using the Neighbor-Joining method according to the sequence alignment of laccases from 16 fungal species with a view to investigating the relationship among these proteins in *P. eryngii* (Fig. 3). The clustering of amino acid sequences in the phylogenetic tree does not strictly follow the phylogeny of the species, were divided into four groups. In Group 1, laccase from *P. eryngii* were clustered with the laccase of *Pleurotus* such as *P. ostreatus*, *P. pulmonarius*, *P. salmoneostramineus*, *P. florida* and also *Lentinus sajor-caju*, which had a high bootstrap value. These fungi in Group 1 were all wood-rooting fungi, and these laccase were late differentiated in fungal laccases. In Group 2, only one laccase of *P. eryngii* (*PeLac8*), has a high degree of homology with *P. ostreatus PoLac12*, clusters with *F. velutipes*, *Lepista nuda*, *C. cinerea*, and *Tricholoma matsutake*. The vast majority of *P. eryngii* laccases were clustered in Group 3 (*PeLac3*, *PeLac4*, *PeLac5*, *PeLac10*), with high bootstrap values with *P. ostreatus*. These laccases are more closely differentiated from *C. cinerea*. Only one *PeLac* (*PeLac9*) clustered with laccase of *V. volvacea*, *F. velutipes* in Group 4.

**Fig. 2:**
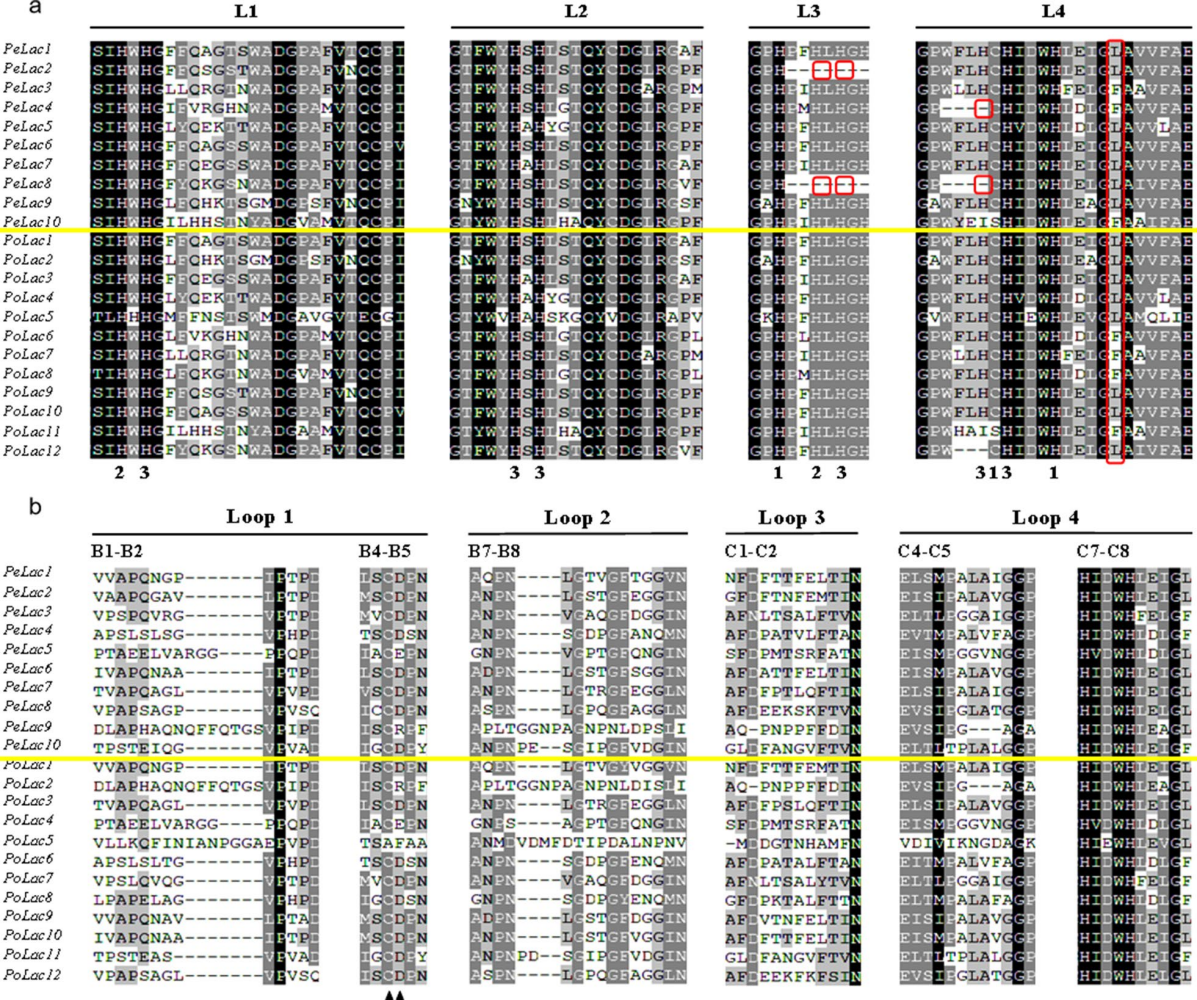
Multiple sequence alignment and structural domain analysis of laccase proteins in *P. eryngii* and *P. ostreatus*. The signature sequences (**a**) and predicted substrate binding loops (**b**) in *P. eryngii Pe*Lac1-10 in comparison with those in *P. ostreatus Po*Lac1-12. The histidine (H) and cysteine (C) residues are numbered according to the type of copper to which they bind (1, 2, and 3). Solid triangles below the sequences denote residues in the B4–B5 β-hairpin loop that are a highly conserved cysteine (C) and either an aspartic acid (D) or a glutamic acid (E) in classical laccases

### Predicted cis-regulatory elements in *PeLac* promoters

Putative cis-regulatory elements within the promoters of *P. eryngii* laccase genes were predicted using the *Arabidopsis thaliana* database in PlantCARE to elucidate possible regulatory patterns of *PeLacs* (Additional file 1: Fig. S3). The cis-regulatory elements were assigned to four main groups: stress-responsive, auxin-responsive, light-responsive, and MYB-binding sites. Apart from *PeLac2*, the remaining 9 *PeLac* promoters contained light-responsive elements, such as G-box (Giuliano et al. 1988; Priest et al. 2009) A total of 4 *PeLacs* (*PeLac2, 6, 7, and 8*) contained anaerobic-responsive elements (ARE) and 7 *PeLacs* (*PeLac1, 3, 4, 5, 6, 7, 8, and 10*) contained low temperature-responsive elements (LTR), with the *PeLac4* promoter possessing a maximum of two. A total of 4 *PeLacs* (*PeLac1, 2, 5, and 7*) contained auxin cis-response elements (AuxRR-core) and all *PeLac* promoters possessed MYB-binding site elements, with the exception of *PeLac7*. Cell-cycle regulatory elements were only found in the *PeLac1* promoter, and one or two salicylic acid-responsive elements were present in the promoters of *PeLac4*, *PeLac9*, and *PeLac10*. Among the 10 *PeLac* promotors, only that of *PeLac3* possessed a defense and stress-responsive element and that of *PeLac7* contained a circadian regulatory element. Taken together, these data show that the type and number of cis-regulatory elements varies among *PeLac* genes and that the specific functions of these cis-regulatory elements require further experimental investigation.

### The structure–function relationship of laccases

Laccases are blue MCOs possessing one type 1 (T1 Cu; blue), one type 2 (T2 Cu; normal), and two type 3 (T3 Cu; coupled binuclear) copper centers (Quintanar et al. 2005). T2 and T3 form a trinuclear cluster that functions to reduce molecular oxygen, releasing water. T1 oxidizes reduced substrates and transfers the resulting electrons to T2 and T3 (Solomon et al. 2008). By modeling the interaction between all 10 laccases and the substrate (such as lignin), which revealed a predominance of beta sheets (Fig. 4). The ɑ-helices of *Pe*Lac3–5 and *Pe*Lac9 exhibited high consistency in the tertiary structure, while those in *Pe*Lac1, *Pe*Lac2, *Pe*Lac6–8, and *Pe*Lac10 were similar. The models show the position of the copper ions, indicating a difference in their number and inter-ion distance. *Pe*Lac7 and *Pe*Lac9 possessed three copper ions (T2 and T3) in the central portion of the protein that failed to be predicted, and their secondary structures were more dispersed in comparison with the other laccases. The presence of histidine deletions at the T2 and T3 copper sites in the L3 conserved sequence (marked by red boxes) in *Pe*Lac2 and *Pe*Lac8 (Fig. 2) also fails to predict the presence of copper atoms in their tertiary structures. Moreover, two copper ions were demonstrated in all other proteins. The secondary structures of all laccases were similar, which indicates high structural and functional consistency. Two T2 Cu were predicted in the tertiary structure of *Pe*Lac5, which are coordinated symmetrically to five histidine-N atoms, providing two channels for molecular oxygen. Their coordination domains can best be described as twisted tetrahedra. Two oxygen atoms are symmetrically coordinated to two histidine residues. This situation is true for the electron transfer and transmission pathway and reflects the mechanism of Cu oxidation and O_2_ reduction (Jones and Solomon 2015).

**Fig. 3:**
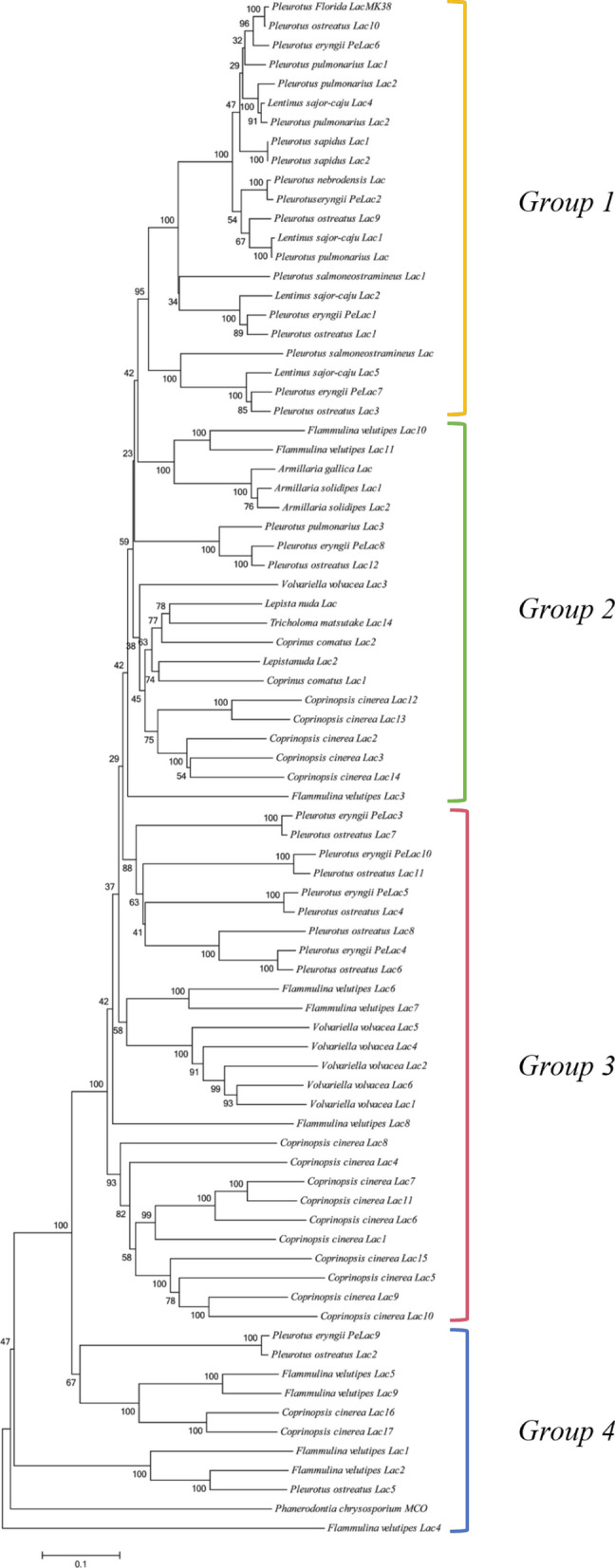
Phylogenetic tree of Lac proteins from *P. eryngii* and 16 other fungi. The Neighbour joining (NJ) method with 1000 ultrafast bootstrap replications using MEGA v7.0. Bootstrap values are from 1000 replications. The scale bar indicates representing 0.2 amino acid substitutions per site

**Fig. 4:**
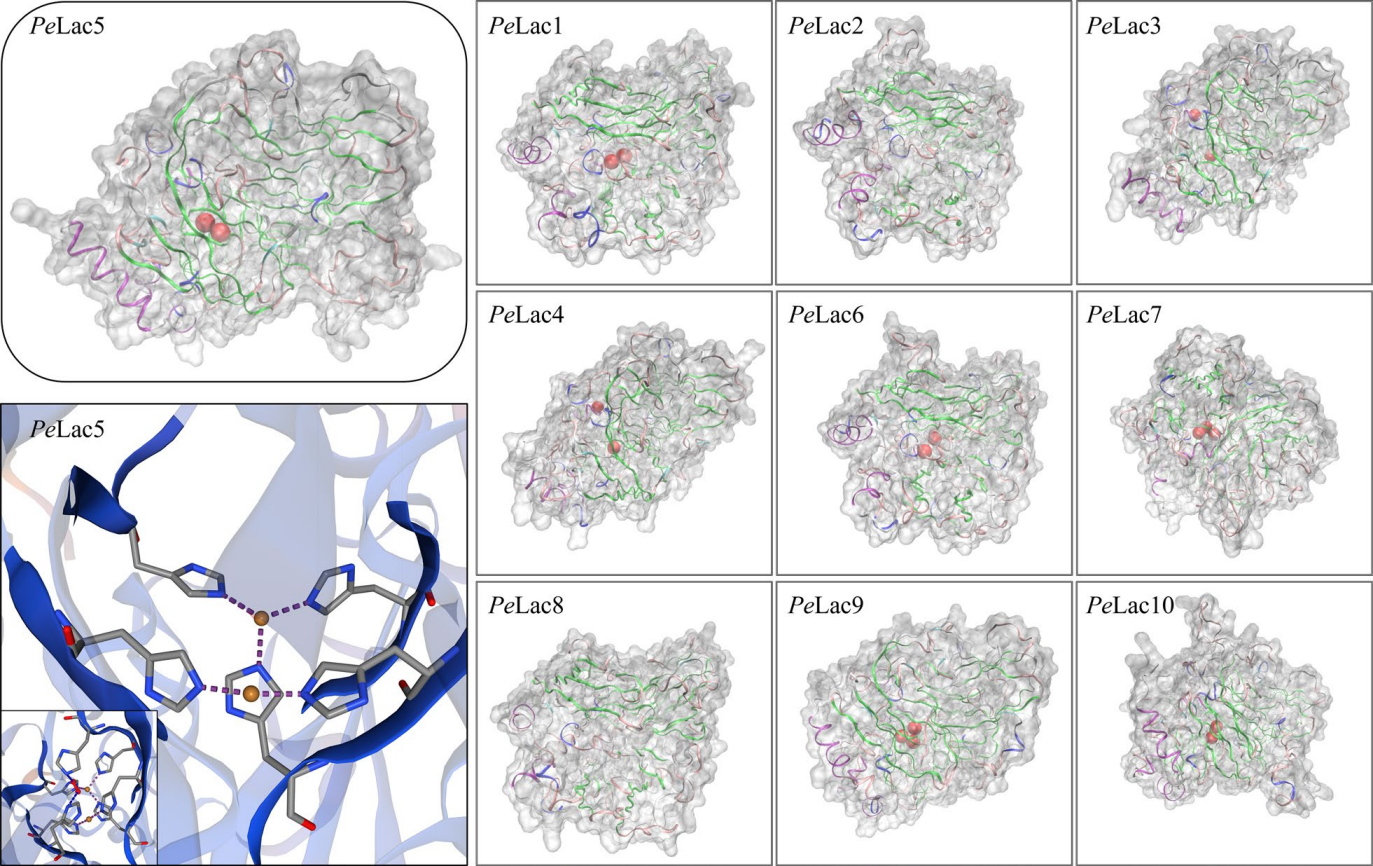
Tertiary structure of *Pe*Lac proteins. All secondary structures are represented by ribbons, with different colors indicating different secondary structures. α-helix, purple; β extended, green; β bridge, cyan; turn, pink; coli, white; 3–10 helix, blue. Copper ions are specified by red spheres. Protein surface is represented by white cloud filling

### Candidate gene selection for verification of function

Previous evaluation of laccase gene expression during different developmental stages has demonstrated that *PeLac5* levels are significantly higher than those of other genes during the mycelium and fruiting stages (Li et al. 2021); therefore, it is important to analyze the function of *PeLac5* in growth and development. Gene silencing by RNAi is a widely used method to assess gene function in fungi (Nakade et al. 2011). To investigate the role of *PeLac5*, overexpression and silencing plasmids containing *hyg* as a selection marker were constructed (Additional file 1: Fig. S1). RT-qPCR revealed that the level of the OE-*PeLac5* transcript was approximately 1.5- and 2.4-fold higher in the OE-*PeLac5*.1 and OE-*PeLac5*.2 strains, respectively, than that in the wild-type (WT) strain, whereas the *PeLac5* transcript was reduced by 47% and 74% in the RNAi-*PeLac5*.17 and RNAi-*PeLac5*.20 strains, respectively (Additional file 1: Fig. S4).

**Fig. 5:**
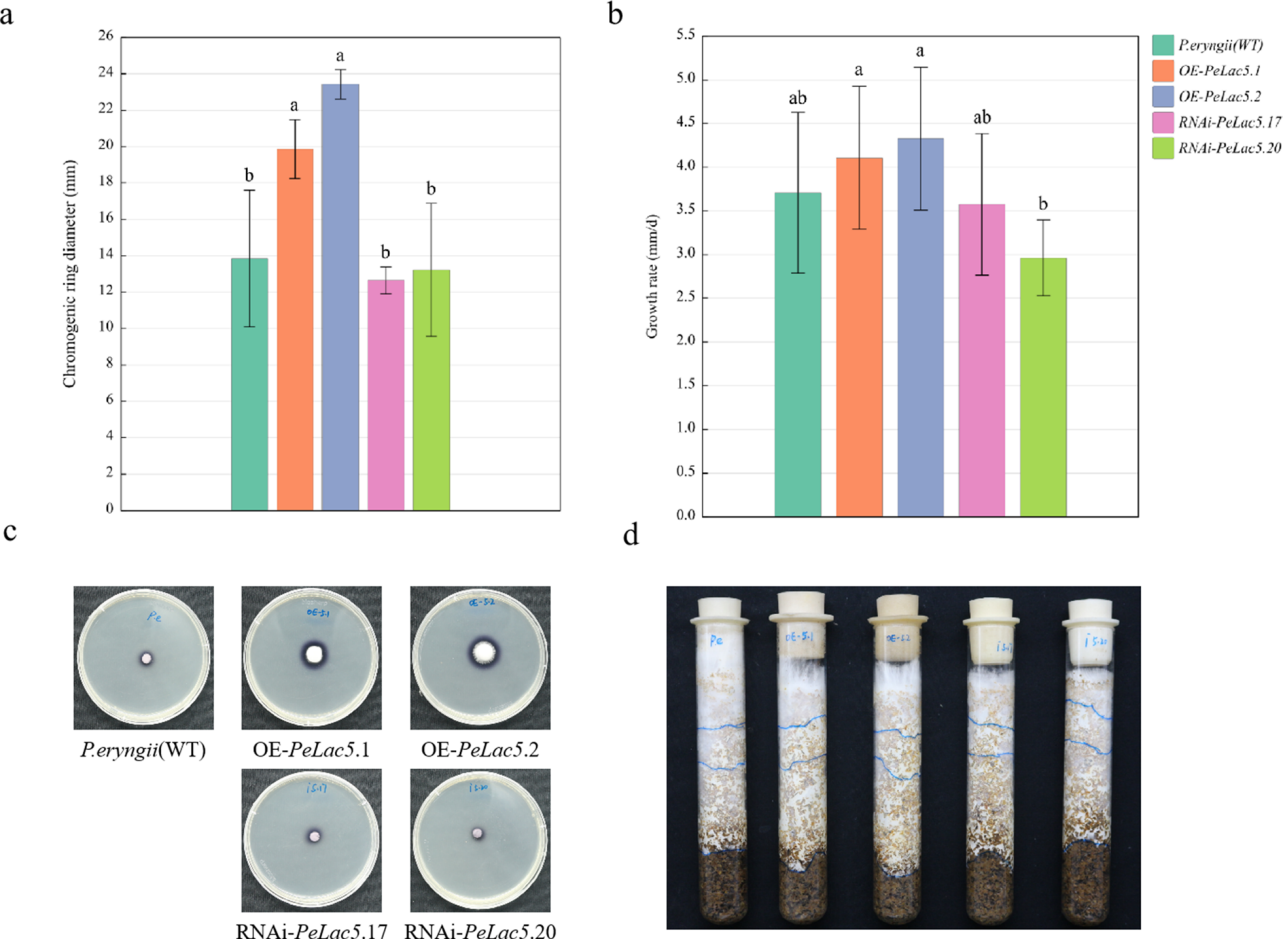
Characterization of the *PeLac5*-overexpressing and -silenced strains. **a**, **c** Diameter of the α-naphthofen colored-halo and statistical analysis. **b**, **d** Mycelial growth rate in the test tube and statistical analysis. The values are the means ± SE of three independent experiments. Different letters indicate significant differences between the transformed strains and the WT strain under 25^o^C cultivation (P < 0.05, according to Duncan’s test)

**Fig. 6:**
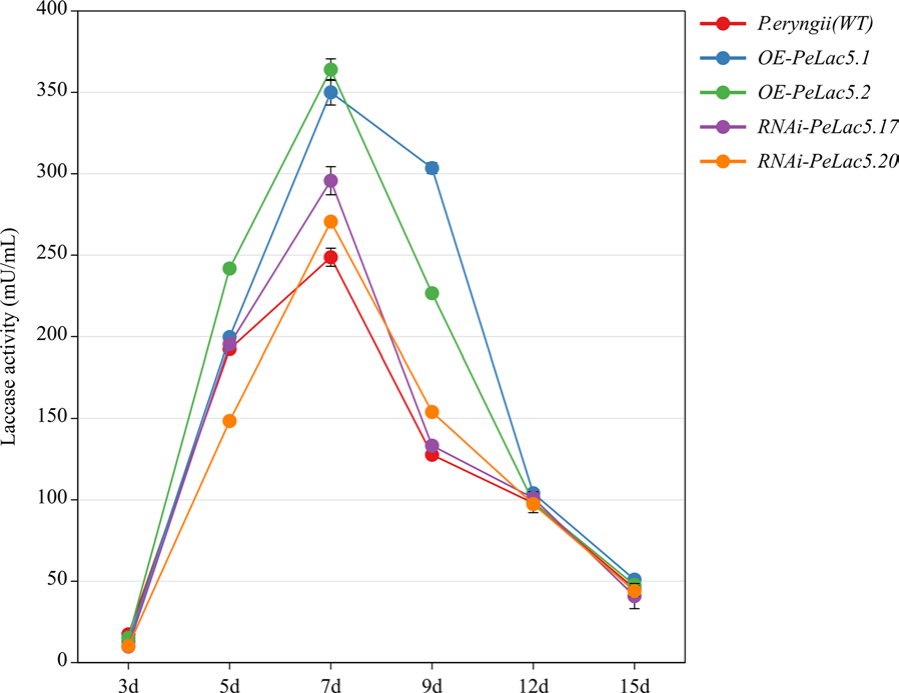
Comparison of the laccase production capacity of wild-type (WT) and *P. eryngii* transformant strains (OE-*PeLac5*.1, OE-*PeLac5*.2, RNAi-*PeLac5*.17 and RNAi-*PeLac5*.20) in CYM liquid medium. The values are the means ± SE of three independent experiments. Different letters indicate significant differences between these strains (P < 0.05, according to Duncan’s test)

To further investigate the effects of *PeLac* overexpression and silencing on mycelial laccase production capacity, incubation and colour development experiments were carried out on wild-type and transformant strains using α-naphtho-PDA plates. In comparison with the WT strain, the *PeLac5-*overexpressing strains exhibited significantly longer colored-halo diameter, whereas the *PeLac5-*silencing strains exhibited shorter colored-halo diameter (Fig. 5a and c). Subsequently, the mycelium growth rate of these overexpressing and silenced strains was measured in a test tube (Fig. 5b and d). The strains overexpressing *PeLac5* grew faster than the WT strain, whereas the silenced strain grew slower. The results of laccase activity assays showed that a maximal value was reached in all strains on day 7 (Fig. 6), with the enzyme activity in strains overexpressing *PeLac5* being 1.4- and 1.5-fold higher, respectively, than that in the WT strain. From day 9, laccase activity began to decrease; however, it remained as much as 1.8- and 2.4-fold higher in the *PeLac5-*overexpressing strains, respectively, than that in the WT strain. However, surprisingly, the enzyme activity in the *PeLac5-*silenced strains was also higher than that in the WT strain on days 7 and 9, respectively, which may be attributed to a compensatory mechanism following the introduction of RNAi.

**Fig. 7:**
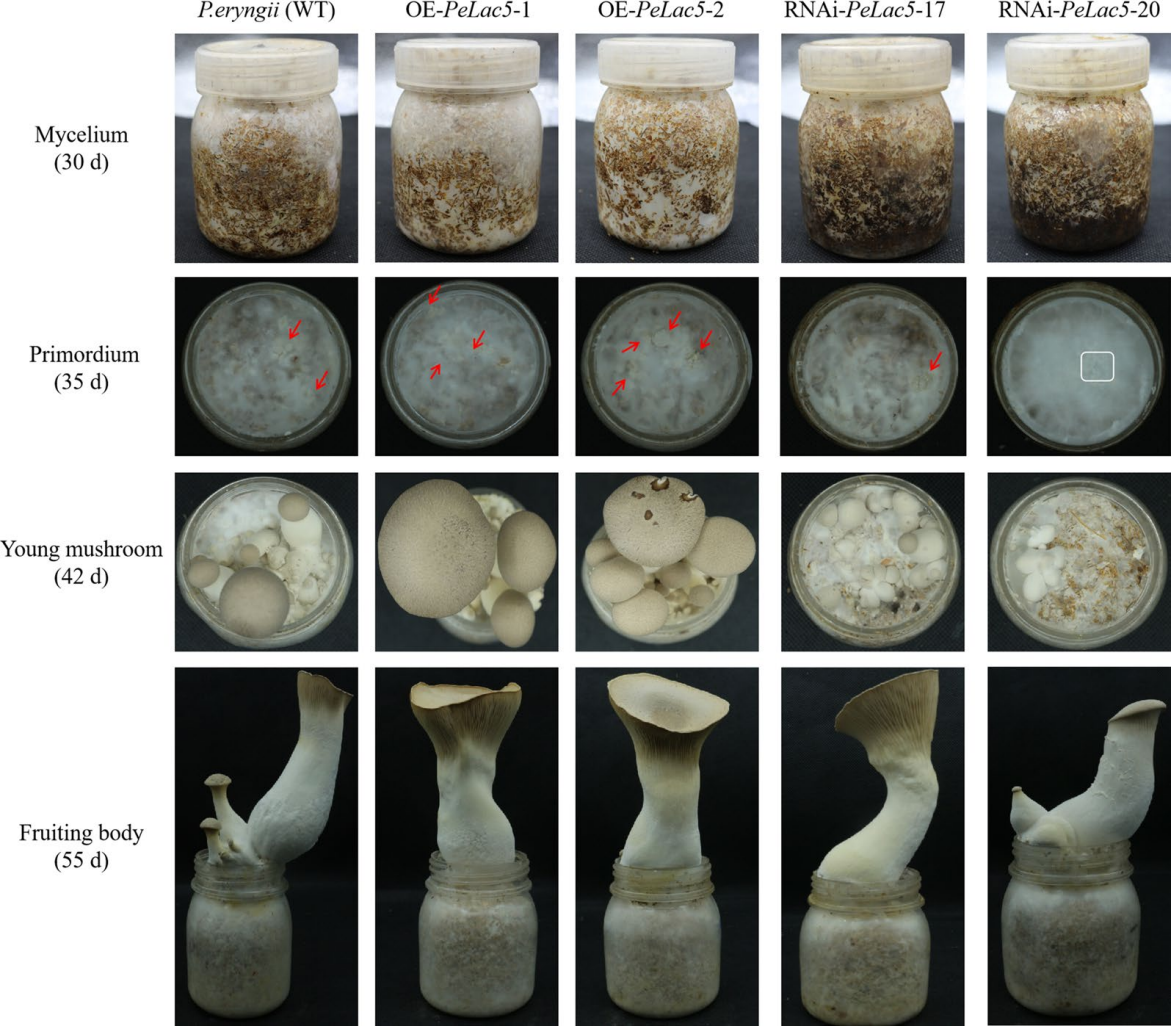
Impact of *PeLac5* on the life cycle (mycelia, primordia, young mushrooms and fruiting bodies) of the *P. eryngii* strains. Each strain was inoculated with 20 cultivated bottles for trait counting

The typical commercial *P. eryngii* cultivar was evaluated with respect to mycelium growth rate, quality, weight, morphological characteristics and cultivation properties. In the mushroom production experiments, primordia were formed in the *PeLac5-*overexpressing strains earlier than those in the WT strain, whereas the *PeLac5-*silenced strains displayed the opposite phenotype. *PeLac5-*overexpressing strains had a more rapid mycelial growth rate than the WT strain during the mycelial period (30 d), and the *PeLac5-*silenced strains showed the opposite phenotype. At the young mushroom stage, the fruiting bodies were significantly larger in the *PeLac5-*overexpressing strains than those in the WT strain, while being dramatically smaller in the *PeLac5-*silenced strains. During the fruiting body period, the stalk diameter of the *PeLac5-*overexpressing strains was larger than that of the WT and *PeLac5-*silenced strains. These data show that *PeLac5* overexpression shortened the period of *P. eryngii* cultivation and *PeLac5* silencing lengthened it (Fig. 7).

### **Expression pattern analysis of *PeLacs *on different lignocellulosic substrates**

**Fig. 8:**
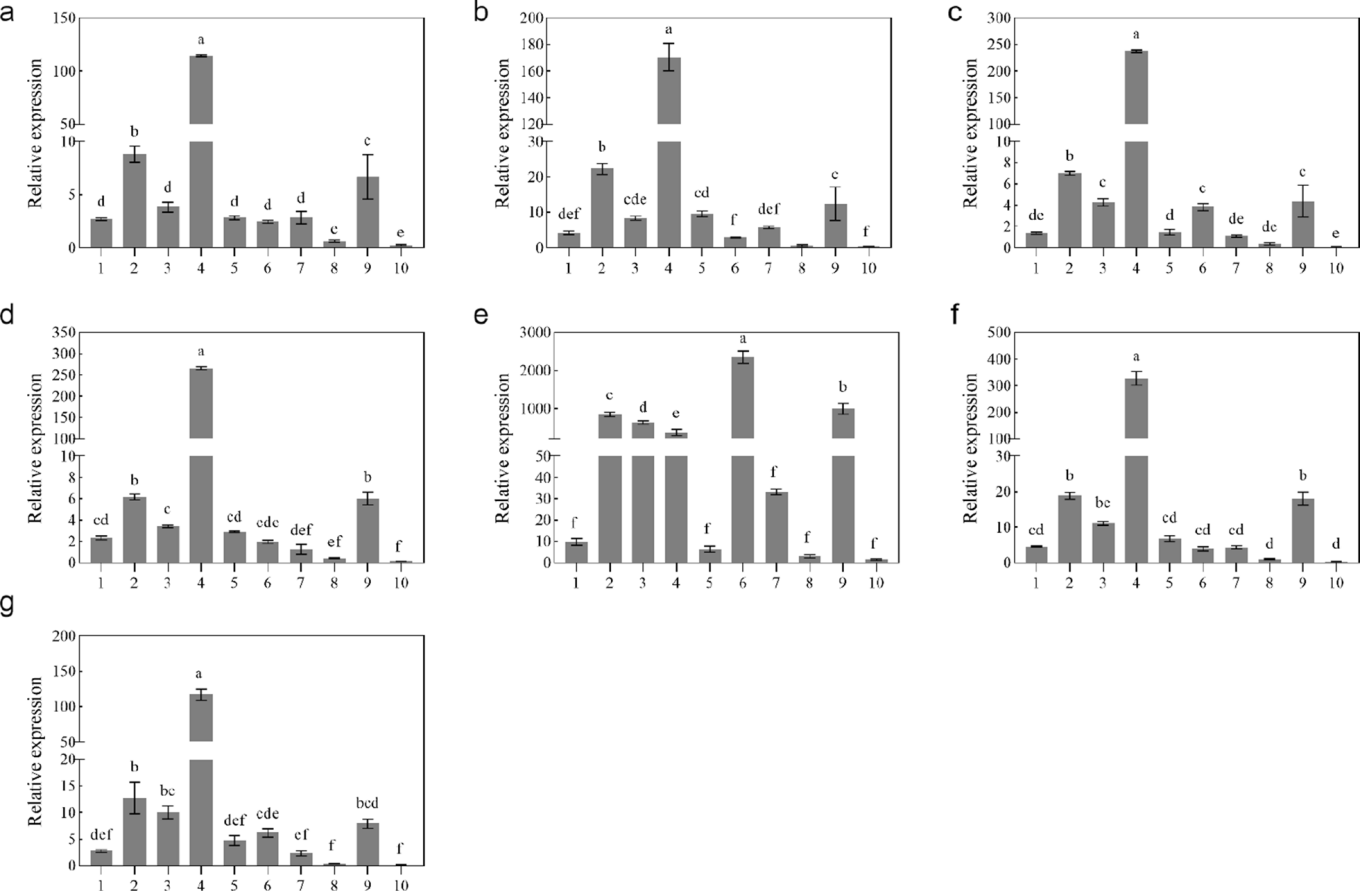
Expression patterns of the *Lac* gene family in *P. eryngii* following growth on different substrates. **a** *Populus simonii*, **b** *Castanea mollissima*, **c** *Pyrus bretschneideri*, **d** *Malus pumila*, **e** *Caragana korshinskii*, **f** *Morus alba, ***g** *Eucalyptus robusta*

To further reveal *PeLac* gene functions, the relative expression patterns of the 10 *PeLac* genes were investigated following cultivation on different lignocellulosic substrates (Fig. 8). The expression patterns of the 10 *PeLac* genes were consistent on the seven natural substrates, *Populus simonii*, *Castanea mollissima*, *Pyrus bretschneideri*, *Malus pumila*, *Caragana korshinskii*, *Morus alba* and *Eucalyptus robusta*. Among the laccase genes, the relative expression of *PeLac4* was the highest (up to 98- to 328-fold) on all substrates except for *Caragana korshinskii*. The other genes with relatively higher expression were *PeLac2*, *PeLac9*, and *PeLac3*. *PeLac6* exhibited the maximum relative expression on *Caragana korshinskii* (2354-fold) but showed no significant differences in expression on the other substrates in comparison with *PeLac1*, *PeLac5*, and *PeLac7*. *PeLac8* and *PeLac10* were hardly or minimally expressed on the seven substrates.

## Discussion

Diversification of laccase genes and the existence of laccase isozymes are frequently seen in a variety of fungi (Janusz et al. 2013). There are 12 laccase genes in the basidiomycetes *Agaricus bisporus*, two of which are located on the same chromosome (Perry et al. 1993; Morin et al. 2012). Increasing availability of fungal genome sequences has stimulated research regarding laccase gene family characterization. In *C. cinerea*, the 17 laccase genes were characterized, and 9 were found to be translated into functional laccases (Kilaru et al. 2006). A total of 12 *PoLacs* have been detected in *P. ostreatus* (Jiao et al. 2018), and 11 laccase genes have been identified in *F. velutipes* (Wang et al. 2015). Here, 10 *PeLac* genes were detected and categorized into three groups, which indicates common diversity of fungal laccase genes and suggests divergence of original laccase genes into paralogs possessing different functions to satisfy particular requirements throughout the fungal life cycle (Kumar et al. 2003).

Analysis of gene structure demonstrated that most *PeLac* genes clustered within the same group had a comparable exon/intron structure (Fig. 1), which is in accordance with previously reported results (Kilaru et al. 2006; Jiao et al. 2018). While different laccase enzymes do not possess identical overall amino acid sequences, their conserved characteristic sequences, named L1–4, show a high degree of consistency (Kumar et al. 2003). The signature L3 sequences in *Pe*Lac2 and *Pe*Lac8 lack the typical two histidine (H) residues, moreover, the first histidine, which is normally present in the T3 Cu centre, is also missing from the signature L4 sequence., which may affect the transfer of electrons during oxidation reactions (Jeuken et al. 2000). The copper atom in the substrate binding site is a T1 Cu and defines the redox potential and thus the substrate range of the enzymes (Liu et al. 2019). The axial ligand for *Pe*Lac1, *Pe*Lac2, and *Pe*Lac5–9 is a leucine (L), predicting a moderate catalytic capacity for these laccases. However, there exists a phenylalanine (F) at this position in *Pe*Lac3, *Pe*Lac4, and *Pe*Lac10, which indicates that these laccases have a high reduction potential and their catalytic capacity may be superior to the other laccases expressed in *P. eryngii*. In *P. ostreatus*, the amino acid residue at this position in *Po*Lac6–8 and *Po*Lac11 is also a phenylalanine (F) (Jiao et al. 2018). These laccases in *P. eryngii* and *P. ostreatus* have a high degree of evolutionary homology (Fig. 3) and it can be speculated that they exert similar functions. This is further supported by the fact that the phylogenetic tree of basidiomycetes laccases does not strictly follow the phylogenetic tree of species but is related to their corresponding functions in the largest enzyme family formed by 125 basidiomycetes laccase species (Hoegger et al. 2006).

The function of proteins is not only related to their evolutionary relationship but is also directly linked to their tertiary structure (Sun et al. 2011; Manavalan et al. 2015; Marisa et al. 2013). For example, *PeLac4* and *PeLac9* were the most highly expressed laccases during substrate degradation, while transcripts of *PeLac8* and *PeLac10* were barely detectable; however, these enzymes exhibited a high degree of similarity in their tertiary structure. Noteworthy, although *PeLac5* is most closely related to *PeLac6* both evolutionarily and with respect to tertiary structure, these enzymes may perform different functions. *PeLac6* is probable for lignocellulose degradation in *P. eryngii* while *PeLac5* appears to be primarily involved in growth and development processes.

Laccases are critical for several physiological activities in fungi, and the existence of multiple laccase isozymes indicates their diverse functions, which include lignin degradation (Hoegger et al. 2006), the formation of fruiting bodies (Chen et al. 2004), the formation of pigment during asexual development (Tsai et al. 1999), and plant pathogenesis (Giardina et al. 2010). Moreover, laccases in mycorrhizal and saprophytic fungi are also involved in the cycling of soil organic matter (Luis et al. 2005). It has previously been shown that laccase isozymes play critical roles in fungal growth and development, performing various specific functions in different species. Lignocellolytic enzymes appear in *P. ostreatus* to be produced for regenerative vegetative hyphal growth (Rühl et al. 2008), *PoLac2* overexpression in *P. ostreatus* enhances its degradation of lignin-rich cotton straw (Jiao et al. 2018). In *F. velutipes*, *lac4* expression is significantly increased during all growth periods, indicating its likely participation in lignin bioconversion (Wang et al. 2015). The growth cycle of edible mushrooms is closely related to laccase activity, with higher activity being associated with shorter cycles (Sun et al. 2011). In the present study, *PeLac5* overexpression resulted in higher laccase activity in comparison with the WT and *PeLac5*-silenced strains from day 5 to day 9. In the mushroom production experiments, *PeLac5-*overexpressing strains also displayed a mycelium growth advantage over the WT and *PeLac5-*silenced strains. Overexpression of *PeLac5* facilitates primordia formation and fruiting body development, in addition to shortening the cultivation cycle. However, the stalk length and fruiting body weight were not statistically different among the three strains despite trend consistency (Additional file 1: Tables S3–S6), which is possibly due to the fact that *PeLac5* is directly involved in morphological development rather than lignin bioconversion. These results are in agreement with reports that *Lcc1* overexpression in *Hypsizygus marmoreus* promotes the growth of mycelia and the priming of fruiting bodies (Zhang et al. 2015).

Laccase genes have also been confirmed to be associated with growth and development in other fungal species. In *Auricularia auricula-judae*, *lcc5* is most highly expressed during the formation and maturation of fruiting bodies, suggesting a principal role in sexual reproduction (Fan et al. 2014). In *F. velutipes*, *lac4* is significantly upregulated in stipe during fruiting body development, suggesting its involvement in their elongation. In *L. edodes*, *Lelcc4* and *Lelcc7* play roles in mycelial browning by promoting the light-induced synthesis of melanin (Yan et al. 2019). Here, our functional studies revealed that overexpression of *PeLac5* resulted in the earlier formation of primordia, which indicates its potential involvement in the regulation of growth and development in *P. eryngii*. Moreover, it is known that organismal development and physiology are controlled by *cis*-regulatory elements via changes in gene activity (Wittkopp and Kalay 2012; Biłas et al. 2016), and those present in laccase genes, including stress-responsive, light-responsive, auxin-responsive, and MYB-binding site *cis*-regulatory elements, may play a role in the regulation of fungal growth and development. A cell-cycle regulatory element was only found in the *PeLac1* promoter and a circadian-cycle regulatory element was only found in the *PeLac7* promoter. Moreover, the promoter region of *PeLac5* contained a light-responsive G-box and a GT1-motif, a low temperature-responsive element (LTR), three auxin-responsive elements (AuxRR-core), a MYB binding site associated with drought inducibility (MBS), and four MYBHv1 binding site elements (CCAAT-box). The fact that the promoter of *PeLac5* in *P. eryngii* contains several *cis*-responsive elements suggests that the encoded proteins perform diverse biological functions involved in fungal growth and development, in addition to responses to biotic and abiotic stresses.

The elucidation of gene expression patterns can provide important insights into gene function (Cao et al. 2016). Here, the relative expression levels of ten *PeLac* genes were evaluated during the mycelial stage of *P. eryngii* cultivated on seven sawdust substrates as the sole carbon source. *PeLac4* displayed the highest expression within the laccase isozyme family on all substrates except *C. korshinskii* (Fig. 8). Moreover, relatively high expression of *PeLac2* and *PeLac9* was seen. These observations indicate that *PeLac4*, *PeLac2*, and *PeLac9* may play decisive roles in the degradation of lignocellulose by *P. eryngii*. Furthermore, *PoLac2*, *PoLac6*, *PoLac9*, and *PoLac10* were confirmed to be essential enzymes in *P. ostreatus* for the decomposition of lignocellulose in sawdust and wheat straw (Fernández-Fueyo et al. 2016). Their evolutionarily similar counterparts in *P. eryngii* were *PeLac9*, *PeLac4*, *PeLac2*, and *PeLac6*, respectively, all of which were instrumental in the degradation of lignocellulose.

Our data also reaffirm the notion that genes with similar evolutionary relationships are functionally identical (Hoegger et al. 2006). The expression of *PeLac8* and *PeLac10* in *P. eryngii* cultivated on these seven substrates was barely detectable, suggesting that these laccases may not be directly involved in the degradation of lignocellulose, which is supported by multiple sequence alignment (Fig. 2). Interestingly, the expression levels of several laccase genes in *P. eryngii* cultivated on *C. korshinskii* were considerably elevated. For example, the relative expression of *PeLac6* reached 2354-fold and others, such as *PeLac2–4*, *PeLac7*, and *PeLac9*, displayed 33–990-fold expression.

Certain laccases, such as *PeLac1* and *PeLac5*, had relative expression levels below 10-fold in *P. eryngii* grown on all seven substrates, indicating their immediate involvement in lignocellulose degradation. It has been reported that *Lacc1* is not directly involved in lignocellulose degradation by *L. edodes*, but instead increases resistance to oxidative stress, which in turn improves degradation of substrates (Cai et al. 2017). In the phylogenetic tree, *PeLac1* clustered with *PoLac1* in group 1, while *PeLac5* clustered with *PoLac4* in group 3, suggesting that the function of *PeLac1* is not identical to that of *PeLac5* despite jointly assisting in the degradation of lignocellulose; therefore, the underlying mechanisms of action of *PeLac1* need to be explored in subsequent experiments.

In summary, our data provide a first glimpse into *P. eryngii PeLacs* and uncover the underlying function of *PeLac5*. *PeLac* genes were characterized with respect to phylogenetic relationships, gene family expression pattern, and gene structure, in addition to protein domain organization and protein tertiary structure. The *cis*-regulatory elements present in their promoters suggest the participation of laccases in fungal growth and development. Furthermore, mushroom growth experiments demonstrated that *PeLac5* was associated with primordia formation and fruiting body development in *P. eryngii*, being positively correlated with fungal growth and development. These findings create a platform from which to further investigate *PeLac* gene function and uncover the effects of laccases on the regulation of fungal development and their degradation of cultivated substrates.

### Supplementary Information

**Additional file 1.** *Pleurotus eryngii* 52,611 genome were deposited into our database and are available at the following URL: http://www.gpgenome.com/species/41180. The accession numbers for the 10 laccase genes are listed in Additional file 1: Table S1. **Fig. S1.** Overexpression and silencing of *PeLac5* in *P. eryngii*. A: Structure of the *PeLac5* overexpression plasmid. B: Structure of the *PeLac5* silencing plasmid. C: Schematic representation of the overexpression and silencing plasmids. Left, *Hyg*R is under the control of the lac promoter; right, *PeLac5*/RNAi is under the control of the *P. ostreatus* *gpd* promoter. D: PCR validation electropherogram of the transformed strains. **Fig. S2.** Amino acid sequence alignment of the conserved domains present within the 10 laccases in *P. eryngii*. Alignment was performed using the Clustal X software. I–III represent the three major conserved domains present in laccase proteins. **Fig. S3.** Cis-element distribution in putative *PeLacs* promoters. **Fig. S4.** qPCR of *PeLac5* expression in the *P. eryngii* wild-type and transformants strains. **Table S1.** Gene accession number. **Table S2.** Primers used in this study. Table S3. The cap diameter of wild-type and transformants. **Table S4.** The stipe length of wild-type and transformants. **Table S5.** The stipe diameter of wild-type and transformants. **Table S6.** Fruiting body weight of wild-type and transformants.

## Data Availability

Available upon the request.
