# Supplementary material for: Genome-wide analysis of the Pleurotus eryngii laccase gene (PeLac) family and functional identification of PeLac5
Source: AMB Express. 2023 Sep 28;13:104. doi: 10.1186/s13568-023-01608-w (PMC10539258; doi:10.1186/s13568-023-01608-w)
Supplement: Supplementary file 1 — Additional file 1. Pleurotus eryngii 52,611 genome were deposited into our database and are available at the following URL: http://www.gpgenome.com/species/41180. The accession numbers for the 10 laccase genes are listed in Additional file 1: Table S1. Fig. S1. Overexpression and silencing of PeLac5 in P. eryngii. A: Structure of the PeLac5 overexpression plasmid. B: Structure of the PeLac5 silencing plasmid. C: Schematic representation of the overexpression and silencing plasmids. Left, HygR is under the control of the lac promoter; right, PeLac5/RNAi is under the control of the P. ostreatus gpd promoter. D: PCR validation electropherogram of the transformed strains. Fig. S2. Amino acid sequence alignment of the conserved domains present within the 10 laccases in P. eryngii. Alignment was performed using the Clustal X software. I–III represent the three major conserved domains present in laccase proteins. Fig. S3. Cis-element distribution in putative PeLacs promoters. Fig. S4. qPCR of PeLac5 expression in the P. eryngii wild-type and transformants strains. Table S1. Gene accession number. Table S2. Primers used in this study. Table S3. The cap diameter of wild-type and transformants. Table S4. The stipe length of wild-type and transformants. Table S5. The stipe diameter of wild-type and transformants. Table S6. Fruiting body weight of wild-type and transformants. [file 13568_2023_1608_MOESM1_ESM.pdf]

AMB Express

Genome-wide analysis of the *Pleurotus eryngii* laccase gene (*PeLac*) family and functional identification of *PeLac5*

Zihao Li[1, 2], Yuanyuan Zhou[1, 2], Congtao Xu[1, 2], Jinlong Pan[1, 2], Haikang Li[1, 2], Yi Zhou[1, 2], Yajie Zou[1, 2]*

[1]State Key Laboratory of Efficient Utilization of Arid and Semi-arid Arable Land in Northern China, Beijing, 100081, China

[2]Institute of Agricultural Resources and Regional Planning, Chinese Academy of Agricultural Sciences, Beijing, 100081, China

*Correspondence:

Yajie Zou

zouyajie@caas.cn, zouyajie8681@126.com

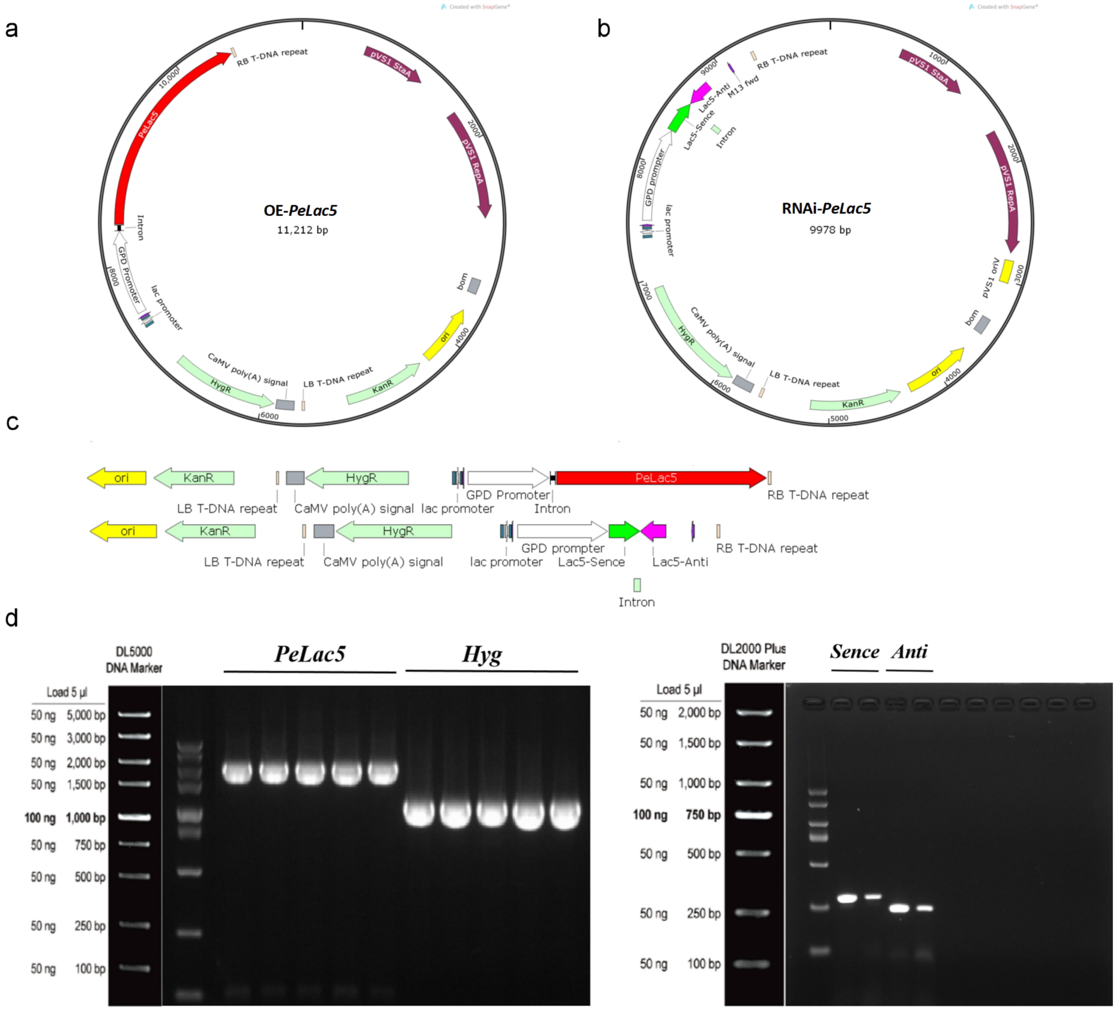

Fig. S1 Overexpression and silencing of *PeLac5* in *P. eryngii*. A: Structure of the *PeLac5* overexpression plasmid. B: Structure of the *PeLac5* silencing plasmid. C: Schematic representation of the overexpression and silencing plasmids. Left, *HygR* is under the control of the *lac* promoter; right, *PeLac5*/RNAi is under the control of the *P. ostreatus gpd* promoter. D: PCR validation electropherogram of the transformed strains.

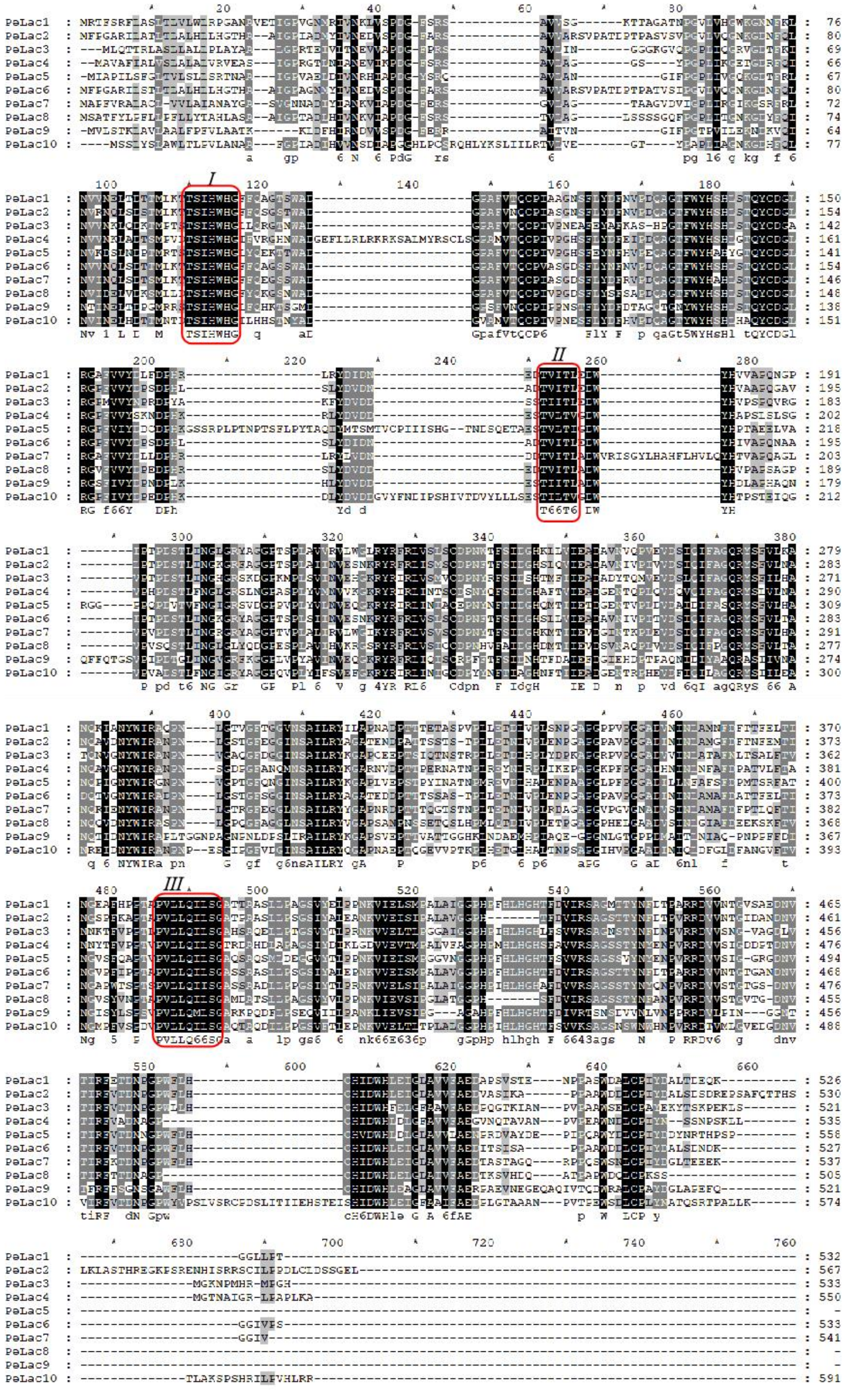

Fig. S2 Amino acid sequence alignment of the conserved domains present within the 10 laccases in *P. eryngii*. Alignment was performed using the Clustal X software. *I–III* represent the three major conserved domains present in laccase proteins.

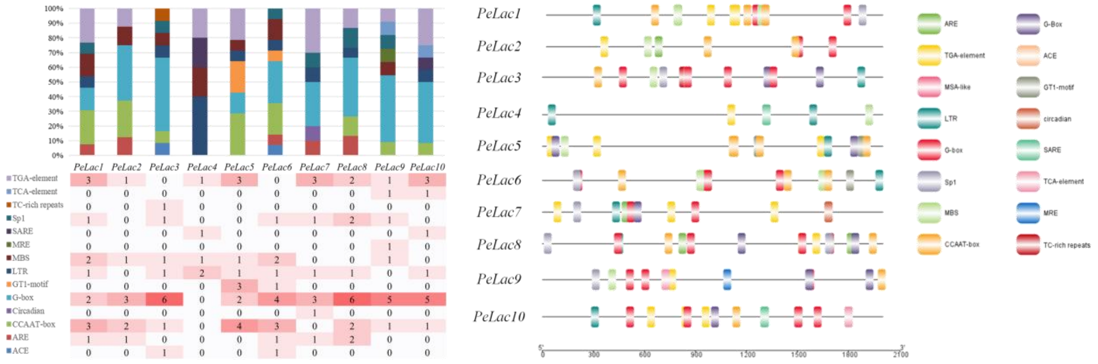

	PeLac1	*PeLac2*	*PeLac3*	*PeLac4*	*PeLac5*	*PeLac6*	*PeLac7*	*PeLac8*	*PeLac9*	*PeLac10*
TGA-element	3	1	0	1	3	0	3	2	1	3
TCA-element	0	0	0	0	0	0	0	0	1	1
TC-rich repeats	0	0	1	0	0	0	0	0	0	0
Sp1	1	0	1	0	0	1	1	2	1	0
SARE	0	0	0	1	0	0	0	0	0	1
MRE	0	0	0	0	0	0	0	0	1	0
MBS	2	1	1	1	1	2	0	0	1	0
LTR	1	0	1	2	1	1	1	1	0	1
GT1-motif	0	0	0	0	3	1	0	0	0	0
G-box	2	3	6	0	2	4	3	6	5	5
Circadian	0	0	0	0	0	0	1	0	0	0
CCAAT-box	3	2	1	0	4	3	0	2	1	1
ARE	1	1	0	0	0	1	1	2	0	0
ACE	0	0	1	0	0	1	0	0	0	0

Fig. S3 *Cis*-element distribution in putative *PeLacs* promoters.

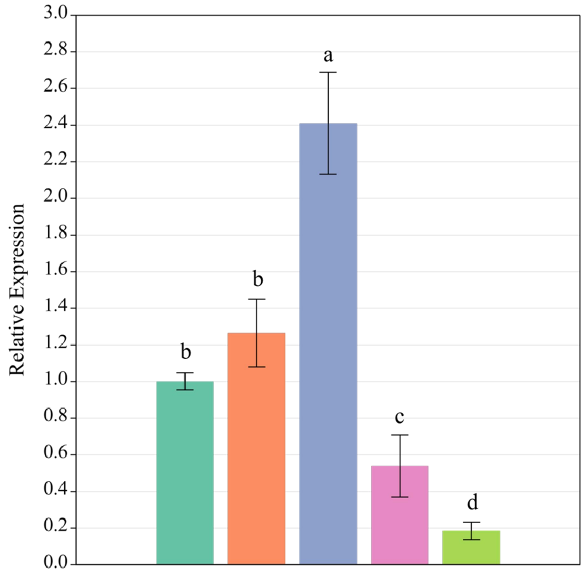

Fig. S4 qPCR of *PeLac5* expression in the 10 *P. eryngii* strains.

Table S1 Gene accession number

Gene name	Accession number
PeLac1	PE_07521-RA
PeLac2	PE_07536-RA
PeLac3	PE_07290-RA
PeLac4	PE_07541-RA
PeLac5	PE_07526-RA
PeLac6	PE_07551-RA
PeLac7	PE_04312-RA
PeLac8	PE_08750-RA
PeLac9	PE_09644-RA
PeLac10	PE_07525-RA

Table S2 Primers used in this study

Primer	Sequence (5′→3′)	Note
PeLac5-F	ATGATTGCGCCTATACTCTCATT	DNA fragment cloning
PeLac5-R	ACCGCACGCATCCTTCGCCCTAA	
PeLac5-OE-F	ttacaggtcaaagttactagtATGATTGCGCCTATACTCTCATTTG	Construction of overexpression plasmids
PeLac5-OE-R	cacgcatccttcgccctaaAAACTATCAGTGTTTGACAGG	
PeLac5-RNAi-F1	ctttaccatctcctcagatctGACGTCACTGTCTTCAACGG	Construction of silencing plasmids
PeLac5-RNAi-R1	ttgaggggtttcaggactagtCTGGTAAGAGTCCTGTCAGA	
PeLac5-RNAi-F2	caggactcttaccagactagtAAGCGAAGATATCAATAGC	
PeLac5-RNAi-R2	gccaattctagagggcccGACGTCACTGTCTTCAACGGTA	
hyg-F	CTCGAGATCCCGGTCGGCATCTACT	Detection of transformants
hyg-F	CCATCTCCGTTTTCTCCCATC	
PeLac5-q-F	GACAATGAGGACCAGTACCAG	RT-qPCR
PeLac5-q-R	GGGACGTGAAAGTTGTACTCG	
Gapdh1-F	AGCAAGGAGATTAGCAAGAG	Endogenous control
Gapdh1-R	CAAGATGAGAGGCGTAGC	

Table S3 The cap diameter of wild-type and transformants

Number	Cap diameter (mm)			significance
P.eryngii	50.13	±	12.55	a
OE-5.1	62.56	±	11.86	a
OE-5.2	59.20	±	7.38	a
OE-5.3	47.55	±	4.65	a
OE-5.4	52.84	±	12.40	a
RNAi-5.10	50.37	±	19.23	a
RNAi-5.17	60.77	±	5.06	a
RNAi-5.20	48.08	±	4.90	a
RNAi-5.23	57.48	±	10.45	a

Table S4 The stipe length of wild-type and transformants

Number	Stipe length (mm)			significance
P.eryngii	69.68	±	14.19	ab
OE-5.1	71.60	±	12.85	ab
OE-5.2	62.63	±	25.87	abc
OE-5.3	75.19	±	12.98	a
OE-5.4	76.12	±	6.40	a
RNAi-5.10	47.18	±	14.83	c
RNAi-5.17	61.81	±	4.93	abc
RNAi-5.20	51.16	±	5.87	bc
RNAi-5.23	73.09	±	2.26	ab

Table S5 The stipe diameter of wild-type and transformants

Number	Stipe diameter (mm)			significance
P.eryngii	33.41	±	8.58	a
OE-5.1	33.79	±	4.97	a
OE-5.2	39.30	±	11.84	a
OE-5.3	37.11	±	5.76	a
OE-5.4	33.89	±	5.46	a
RNAi-5.10	30.31	±	3.55	a
RNAi-5.17	30.65	±	4.71	a
RNAi-5.20	34.84	±	4.92	a
RNAi-5.23	33.36	±	3.51	a

Table S6 Fruiting body weight of wild-type and transformants

Number	Fruiting body weight (g)			significance
P.eryngii	44.59	±	7.01	a
OE-5.1	38.11	±	5.22	a
OE-5.2	41.86	±	7.89	a
OE-5.3	41.58	±	3.74	a
OE-5.4	42.60	±	2.06	a
RNAi-5.10	40.87	±	10.84	a
RNAi-5.17	46.46	±	3.53	a
RNAi-5.20	36.32	±	2.90	a
RNAi-5.23	36.86	±	5.28	a
